# Assurer la continuité des soins au temps de la COVID-19 : défis pour le centre de traitement des addictions à Dakar

**DOI:** 10.48327/V3RB-PV49

**Published:** 2021-02-18

**Authors:** I. Ba, A. Desclaux, M. Diop, I. Ndiaye, M.H. Thiam

**Affiliations:** 1 Centre de prise en charge intégrée des addictions de Dakar (CEPIAD), Sénégal; 2 Université Cheikh Anta Diop de Dakar, Sénégal; 3 Centre régional de recherche et de formation à la prise en charge du VIH et des maladies infectieuses (CRCF), CHUN de Fann, Dakar, Sénégal; 4 Institut de recherche pour le développement, TransVIHMI, Dakar, Sénégal; 5 Projet CODISOCS: Consommateurs de drogues injectables et dynamiques sociales au Sénégal, ANRS 12383; 6 Service de psychiatrie, Centre hospitalier national universitaire de Fann (CHNUF), Dakar, Sénégal

**Keywords:** COVID-19, Usagers de drogues, Services de santé, Impact socio-sanitaire, Sénégal, COVID-19, Drug users, Health services, Socio-sanitary impact, Senegal

## Abstract

La pandémie de COVID-19 a eu un impact sur le fonctionnement des systèmes de santé dont les modes d’adaptation et de réponse sur le terrain sont encore peu documentés et en cours d’évolution. Le Centre de Prise en charge Intégrée des Addictions de Dakar (CEPIAD) met en œuvre depuis 2014 la réduction des risques auprès d’usagers de drogues. La pandémie de COVID-19 et les mesures de santé publique ont été un obstacle à sa fréquentation par les patients, notamment du fait de la limitation des déplacements. Outre la mise en place de mesures préventives et l’application des gestes barrières dans le centre, le CEPIAD a eu recours à l’emport à domicile de la méthadone habituellement dispensée chaque jour dans ses locaux. Le centre a aussi pris en charge des usagers de cannabis incarcérés après leur amnistie. Plusieurs aspects de l’expérience du CEPIAD, perçue positivement, pourraient être pertinents hors du contexte pandémique.

## Abstract (en)

The COVID-19 pandemic has an impact on health systems, whose modes of adaptation and response on the ground are still poorly documented and are evolving. The Dakar Integrated Support Center for Addictions (CEPIAD) has been implementing risk reduction since 2014, particularly with drug users. The COVID-19 pandemic and related public health measures were an obstacle to its attendance by patients, in particular due to movement restriction. In addition to the implementation of individual and collective preventive measures in the center, CEPIAD has experimented “take-home” for methadon that is generally provided daily through directly observed treatment. The center has also taken care of amnestied incarcerated cannabis users. Several aspects of this experience, perceived positively, could be relevant outside the pandemic context.

## Introduction

L’Afrique a subi les effets de la COVID-19 à une moindre échelle que d’autres continents pour ce qui concerne la mortalité enregistrée, pour des raisons multiples: réponse précoce des États, structure démographique, environnement, mode de vie, etc… [9], mais la « seconde vague » survenue en fin 2020 met à mal les systèmes de soins [3]. Les mesures de santé publique mises en place pour lutter contre la pandémie (interdiction des rassemblements, confinement, restriction des déplacements, etc.) ont eu un impact socio-sanitaire notable, plus difficile à mesurer car indirect, multiforme et à plus long terme. Les effets indirects sur la prise en charge d’autres pathologies et sur les interventions de santé publique sont une préoccupation constante, justifiée par les ruptures d’accès aux antirétroviraux et la suspension des programmes de vaccination observées depuis mars 2020 [1]. Parmi les interventions de santé, celles qui requièrent la présence des bénéficiaires dans les formations sanitaires sont particulièrement sensibles à un impact négatif de la pandémie. C’est le cas pour le traitement des addictions au CEPIAD, qui apporte des soins quotidiens aux usagers de drogues de la région de Dakar.

**Fig. 1:**
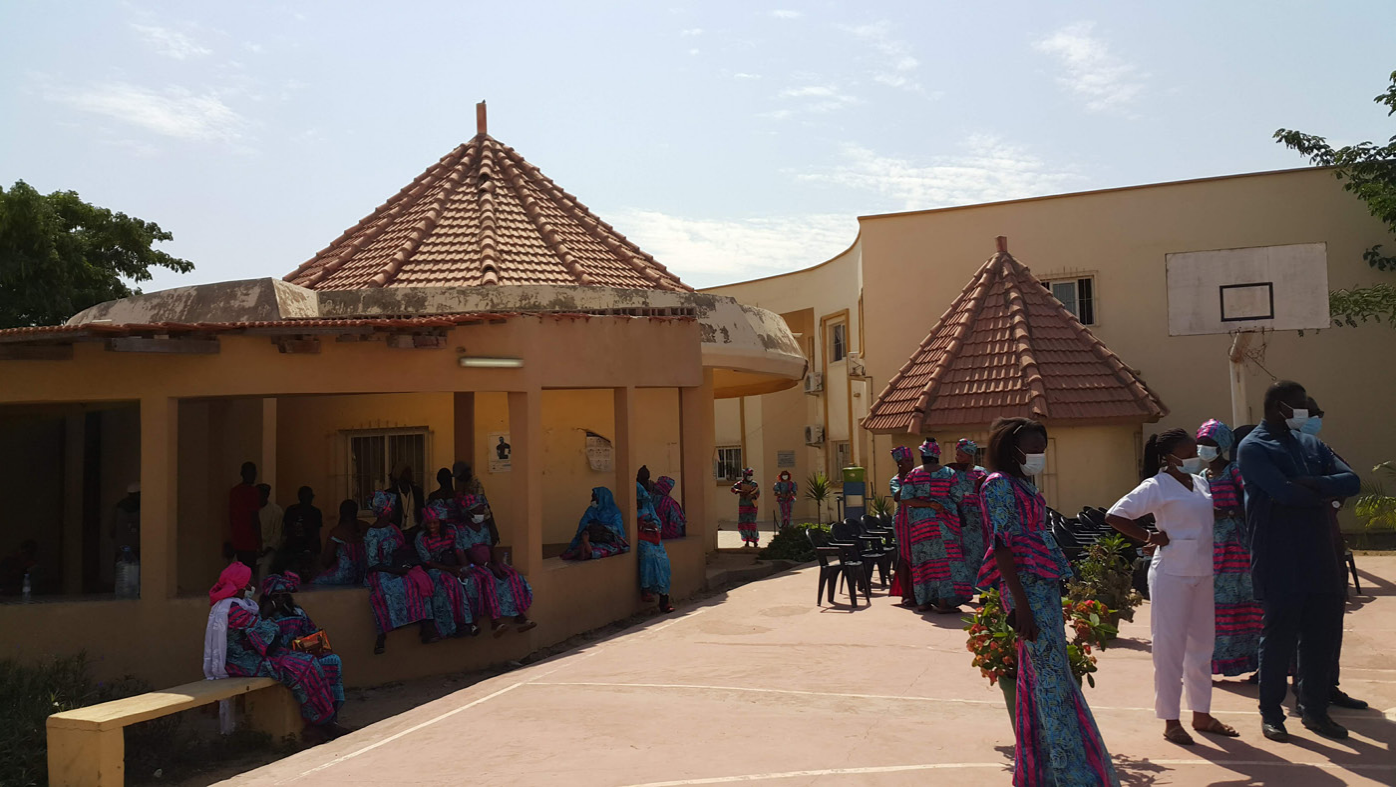
Le CEPIAD lors de la célébration de la Journée Mondiale contre le Sida, le 1^er^ décembre 2020 © Alice Desclaux CEPIAD at the celebration of World AIDS Day, December 1, 2020 © Alice Desclaux

## Le cepiad

Le Centre de Prise en charge Intégrée des Addictions de Dakar (CEPIAD) a été ouvert en 2014, après qu’une enquête épidémiologique ait estimé à 1 324 le nombre d’usagers de drogues injectables dans la région administrative de Dakar [6]. Dans un pays où l’épidémie de VIH touche essentiellement les populations clés, avec un taux de prévalence de 0,7 % dans la population générale alors que celui du VHC était de 0,49 %, la création de ce centre offrant à la fois soins et prévention apparaissait comme une intervention prioritaire pour la lutte contre le sida et l’hépatite C. De plus, le centre allait répondre à une attente forte des usagers ayant peu accès aux structures de soins du fait de leur marginalité sociale [2]. Grâce à l’engagement d’acteurs institutionnels de la direction du Ministère de la santé et de l’action sociale et du Conseil national de lutte contre le sida ainsi que de médecins psychiatres du service de psychiatrie du Centre hospitalier national universitaire de Fann, à l’appui du Fonds Mondial et de l’Organisation des nations unies contre les drogues et le crime (ONUDC), et en concertation avec les associations d’usagers, le CEPIAD a été le premier centre de traitement des adductions qui développe la stratégie de Réduction des Risques (RdR) en Afrique de l’Ouest francophone.

La stratégie de Réduction des Risques (RdR, également appelée « stratégie de réduction des méfaits ») est reconnue au niveau international comme l’approche la plus efficace en termes de santé publique et la plus respectueuse des droits des personnes atteintes d’addiction pour répondre à leurs besoins socio-sanitaires, prévenir les risques infectieux majeurs chez les usagers de drogues (VIH, hépatites, tuberculose multi-résistante) et lutter contre les méfaits des drogues [8,10]. Développée depuis une trentaine d’années d’abord dans les pays du Nord dans le contexte de la lutte contre le sida, la RdR est mise en œuvre dans un nombre de pays croissant, notamment en Afrique à la suite de projets pilotes (Tanzanie, Afrique du Sud, Maroc). Plutôt que chercher à interrompre toute consommation de stupéfiant, ce qui n’empêche ni les effets neuropsychologiques éprouvants d’un sevrage brutal ni les rechutes, la RdR procède de manière globale et progressive en informant les usagers sur les risques qu’ils encourent, en réduisant les pratiques à risques d’infection, en substituant aux opiacés illicites des produits licites délivrés sous contrôle médical, et en considérant les dimensions psychologiques et sociales de l’addiction. Cette approche combine la prise en compte globale de l’addiction (comme rencontre entre un produit, une personne et un contexte) et des connaissances scientifiques en neurobiologie développées au cours des dernières décennies. À Dakar, le CEPIAD est le premier centre de la région Afrique de l’ouest et du centre (AOC) francophone qui propose aux usagers de drogues une prise en charge pour les stupéfiants (en incluant la méthadone pour les personnes dépendantes de l’héroïne), pour le tabac et l’alcool.

Depuis son ouverture, le CEPIAD a pris en charge 2004 patients usagers de drogues (au 31/12/20). Son intégration dans le service de psychiatrie du CHNU de Fann et la collaboration avec le service de maladies infectieuses et le projet de recherche et d’intervention CODISEN^1^, ont permis de proposer un diagnostic et une prise en charge pour l’infection à VIH, la tuberculose, les hépatites A et B, et les infections sexuellement transmissibles, en plus des problèmes de santé mentale. De plus, une équipe outreach (menant des activités dans les communautés) rattachée au CEPIAD développe des interventions d’information sur les risques, de distribution de matériel de prévention (préservatifs, échange de seringues) et de suivi des usagers dans les quartiers de Dakar. Des médiateurs pairs renforcent le lien avec les usagers notamment au travers d’associations.

**Fig. 2:**
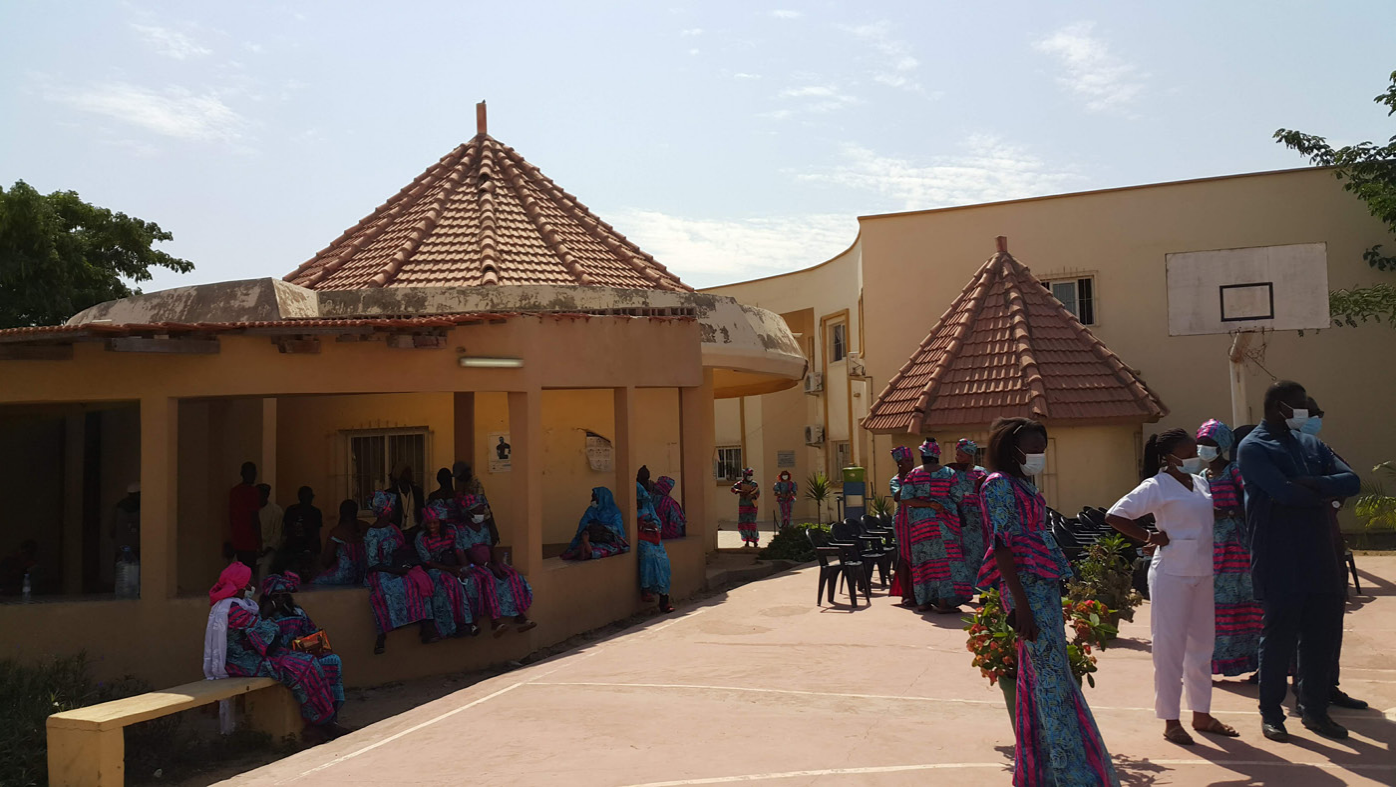
Rencontre de l’équipe soignante avec les usagers au CEPIAD ©Idrissa Ba Health care team meeting with users at CEPIAD ©Idrissa Ba

## Les dÉfis au temps de la covid-19

Le Sénégal a été le second pays d’Afrique sub-saharienne à enregistrer des cas de COVID-19 à partir du 2 mars 2020. Mais c’est à partir du cinquième cas, identifié le 10 mars à Touba dans un service de soins, à l’origine d’un cluster de 21 personnes, que l’épidémie s’est progressivement étendue. À partir de mars 2020, le CEPIAD a décidé de procéder à une régulation de la fréquentation des usagers dans l’objectif de prévenir la transmission du coronavirus au centre. En temps ordinaire, la grande majorité des usagers de drogues injectables traités par méthadone sont astreints à une prise quotidienne de leur traitement au CEPIAD, selon le principe du « traitement directement observé ». Ceci permet d’une part d’être certain que ces patients reçoivent le traitement dont ils ont besoin à la dose prescrite, d’autre part d’éviter que ce traitement soit détourné vers le marché illicite des stupéfiants, ce que la forme sirop prévient également. La venue quotidienne au centre permet aussi aux personnes de maintenir la relation avec l’équipe de soin et de participer à des activités qui contribuent à leur re-socialisation (groupes de parole, causeries, art-thérapie, jardinage, club méthadone).

Mais les rassemblements ont été interdits mi-mars, puis l’état d’urgence a été décrété fin mars avec un ensemble de mesures de distanciation qui ont provoqué une diminution du nombre de places dans les transports en commun et une augmentation du coût pour les passagers; après un allégement pendant plusieurs mois, ces mesures ont été réinstaurées fin 2020. Ces mesures ont eu un impact sur les possibilités de déplacement des usagers du centre, en particulier ceux habitant dans la banlieue populaire de Pikine. Parallèlement à ces difficultés d’accès observées au CEPIAD, la nécessité pour l’équipe outreach de disposer de matériel de prévention (gel hydroalcoolique, gants et masques) a imposé la suspension des sorties sur le terrain. Ainsi, le CEPIAD a d’abord été confronté aux mêmes défis que de nombreux services de soins: comme pour d’autres services hospitaliers, les patients ont déserté le service par défaut d’accessibilité ou par crainte d’être contaminés; comme pour d’autres programmes d’intervention en communauté, les activités hors du centre ont été freinées par la nécessité de disposer de matériel spécifique et la crainte d’un risque « invisible » lorsque les cas communautaires sont devenus plus nombreux.

Un défi spécifique a été la prise en charge des usagers incarcérés qui ont bénéficié le 26 mars d’une grâce présidentielle d’une ampleur destinée à réduire la surpopulation carcérale pour prévenir la propagation du coronavirus. Mise en œuvre dans de nombreux pays selon des modalités variées [7], cette mesure a concerné au Sénégal 2036 détenus, sélectionnés parce qu’ils purgeaient des peines courtes pour des délits mineurs, appartenaient aux tranches d’âge à risque de complications de la COVID-19, ou étaient proches de la fin de leur peine. Un grand nombre d’entre eux étaient des usagers de cannabis: au Sénégal, la détention, l’usage et le trafic de drogues motivent environ une incarcération sur trois. Parmi les personnes libérées, sept sont venues de leur propre chef consulter au CEPIAD, où elles avaient déjà été prises en charge avant leur incarcération. Cette expérience a illustré l’intérêt qu’aurait eu l’orientation vers le CEPIAD de tous les usagers de drogues libérés, alors que l’injonction thérapeutique, une modalité présente dans la loi sénégalaise mais très rarement mise en pratique jusqu’à présent, doit être redéfinie et activée.

## Les adaptations pour assurer les soins

Dès le 7 mars 2020, des dispositions pour combiner la continuité des soins et la mise en pratique de la prévention ont été définies lors d’une réunion du personnel du CEPIAD. Ces dispositions, en phase avec les recommandations de l’ONUDC (UNODC, 2020), visaient à espacer et contrôler les interactions entre équipe soignante et usagers tout en assurant les soins, et à généraliser l’application de distances de sécurité et des gestes barrière, avec un port du masque rendu obligatoire dans les lieux publics par décret ministériel le 19 avril. La réduction des interactions dans le centre a reposé essentiellement sur une redéfinition du planning des consultations de chaque soignant désormais uniquement sur rendez-vous, et la suppression des activités de groupe. De plus, une circulation des patients dans le centre (circuit, durée, lieux de stationnement) a été définie qui ne permet pas aux usagers de rester plus de temps que nécessaire pour collecter leur méthadone et si besoin réaliser leur consultation. La socialisation informelle des patients dans divers lieux du centre (espace de sociabilité, espaces d’attente, atelier, jardin) est désormais évitée et la distance de sécurité qui doit être respectée dans les files d’attente est matérialisée par un marquage au sol. Un dispositif de lavage des mains a été installé à l’entrée et l’application des mesures, notamment le port du masque et le respect des distances de sécurité, est vérifiée par un vigile.

Une mesure particulière au CEPIAD est l’emport de la méthadone à domicile généralisé qui a pour objectif d’éviter d’accroître le risque COVID collectif lié à une fréquentation du centre par un grand nombre de personnes venues de lieux divers, tout en favorisant l’observance grâce à une amélioration de l’accès au traitement. Cette stratégie avait déjà été testée puis progressivement mise en place pour des usagers sous méthadone observants et stables cliniquement, inclus dans le programme depuis septembre 2016, et qui ne pratiquaient pas de polyconsommation en parallèle à la méthadone [4]. Au plan international, l’emport du traitement de substitution aux opiacés est peu développé dans les centres d’addictologie car un traitement est souvent disponible auprès des médecins de ville, ce qui n’est pas le cas au Sénégal où les interactions quotidiennes au centre sont privilégiées pour resocialiser les usagers. À partir de mars, l’emport a été proposé à tous les patients pour des périodes allant de trois jours à une semaine. Le bilan de cette expérience montre que, malgré les risques de détournement et de mésusage de la méthadone soulevés, le dispositif mis en place a été bien accepté et bien suivi. Très peu de problèmes ont été notés (un cas d’overdose à la méthadone chez un usager non suivi au CEPIAD y a été reçu et traité par naloxone). Malgré la très forte exposition des usagers de l’hôpital qui abrite une unité de traitement de la COVID-19, aucun cas de COVID-19 n’a été enregistré au niveau du CEPIAD en 2020, alors que dans le même temps des cas ont été signalés au service de psychiatrie, au niveau des patients et du personnel soignant et d’accueil. Fin 2020, la stratégie de prévention de la COVID-19 combinée au maintien des services aux patients s’est donc montrée efficace et satisfaisante.

## La pandÉmie de covid-19, et aprÈs?

Plusieurs mesures adoptées pour répondre en urgence à la menace infectieuse représentée par la pandémie de COVID-19 concernent des questions débattues au Sénégal, comme au plan international. Ainsi, la situation dans les prisons a rappelé l’intérêt des alternatives à l’incarcération, porteuse de risques sanitaires pour les usagers incarcérés. Par ailleurs, l’application à plus large échelle de l’emport à domicile de la méthadone pendant l’épidémie de COVID-19 et l’analyse de ses effets pourraient permettre d’évaluer le rapport entre avantages et risques de cette mesure, qui rend le traitement moins contraignant et autorise une activité professionnelle facilitant la réinsertion sociale. Plus globalement, ces adaptations vont dans le sens d’une approche des usagers de drogues qui favorise les soins et la prévention plutôt que la seule répression au travers de la pénalisation, en phase avec la dépénalisation promue par la Commission Globale de Politique en Matière de Drogues au niveau mondial [5]. Si cette approche pouvait être maintenue au-delà de la phase de crise épidémique et renforcée, la pandémie de COVID-19 aura été une opportunité pour une avancée sur le plan de la santé publique et des droits humains.

## Remerciements

À l’équipe du CEPIAD et à ses patients. À l’ANRS Maladies Infectieuses Émergentes, pour le financement des projets ANRS 12334 et 12383.

## Conflits D'intérêts

Les auteurs ne déclarent aucun conflit d’intérêts.
